# Alpha-Ketoglutarate: An Effective Feed Supplement in Improving Bone Metabolism and Muscle Quality of Laying Hens: A Preliminary Study

**DOI:** 10.3390/ani10122420

**Published:** 2020-12-17

**Authors:** Ewa Tomaszewska, Sylwester Świątkiewicz, Anna Arczewska-Włosek, Dorota Wojtysiak, Piotr Dobrowolski, Piotr Domaradzki, Izabela Świetlicka, Janine Donaldson, Monika Hułas-Stasiak, Siemowit Muszyński

**Affiliations:** 1 Department of Animal Physiology, Faculty of Veterinary Medicine, University of Life Sciences in Lublin, Akademicka St. 12, 20-950 Lublin, Poland; 2 Department of Animal Nutrition and Feed Science, National Research Institute of Animal Production, Krakowska St. 1, 32-083 Balice, Poland; sylwester.swiatkiewicz@izoo.krakow.pl (S.Ś.); anna.arczewska@izoo.krakow.pl (A.A.-W.); 3 Department of Animal Genetics, Breeding and Ethology, Faculty of Animal Sciences, University of Agriculture in Kraków, 24/28 Mickiewicza Ave., 30-059 Cracow, Poland; wojtysiakd@wp.pl; 4 Department of Functional Anatomy and Cytobiology, Faculty of Biology and Biotechnology, Maria Curie-Sklodowska University, Akademicka St. 19, 20-033 Lublin, Poland; piotr.dobrowolski@umcs.lublin.pl (P.D.); monhul@o2.pl (M.H.-S.); 5 Department of Commodity Science and Processing of Raw Animal Materials, Faculty of Animal Sciences and Bioeconomy, University of Life Sciences in Lublin, Akademicka St. 13, 20-950 Lublin, Poland; piotr.domaradzki@up.lublin.pl; 6 Department of Biophysics, Faculty of Environmental Biology, University of Life Sciences in Lublin, Akademicka St. 13, 20-950 Lublin, Poland; izabela.swietlicka@up.lublin.pl; 7 School of Physiology, Faculty of Health Sciences, University of the Witwatersrand, 7 York Road, Parktown, Johannesburg 2193, South Africa; janine.donaldson@wits.ac.za

**Keywords:** α-Ketoglutaric acid, AKG, laying hens, bone, intestine, hormone

## Abstract

**Simple Summary:**

High egg production in laying hens can negatively affect their skeletal system, which serves as a reservoir of minerals. Intense bone metabolism due to eggshell production in laying hens can lead to bone loss and subsequent fractures. Spent laying hens’ meat can be also used as raw material for the manufacture of processed products. Alpha-ketoglutarate (AKG), a precursor of amino-acid glutamine, effectively improves growth performance, bone metabolism, immunity, and alleviate intestinal mucosal damage. The aim of this experiment was to assess the effect of dietary AKG supplementation on performance, serum hormonal parameters, intestine structure, bone parameters, articular cartilage degradation and characteristics of meat quality of laying hens with a mature skeletal system. The results of our study showed that dietary AKG supplementation did not influence feed intake, weight gain, or laying performance, but improved bone metabolism, increased bone collagen synthesis. Moreover, dietary AKG significantly decreased the cholesterol content of breast muscle. The results showed that AKG can be a valuable feed supplement, positively influencing the bone health status and welfare of laying hens.

**Abstract:**

The aim of the experiment was to assess the effect of dietary alpha-ketoglutarate (AKG) supplementation on performance, serum hormonal indices, duodenum and jejunum histomorphometry, meat quality characteristics, bone quality traits and cartilage degradation in laying hens with a mature skeletal system. Forty-eight 30 week-old Bovans Brown laying hens were randomly assigned to a control group or the group fed the basal diet plus 1.0% AKG. The experimental trial lasted 30 weeks. The supplementation of AKG increases blood serum content of leptin, ghrelin, bone alkaline phosphatate and receptor activator of nuclear factor kappa-Β ligand, while osteoprotegerin and osteocalcin decrease. While dietary AKG was given to laying hens negatively influenced villus length, crypt depth, villus/crypt ratio and absorptive surface area in duodenum and jejunum, these changes have no effect on feed intake, weight gain, nor laying performance. In breast muscles, no significant changes in skeletal muscle fatty acid composition were observed, however, a higher shear force and decreased cholesterol content following AKG supplementation were noted, showing the improvement of muscle quality. While dietary AKG supplementation did not affect the general geometric and mechanical properties of the tibia, it increased collagen synthesis and enhanced immature collagen content. In medullary bone, an increase of bone volume fraction, trabecular thickness, fractal dimension and decrease of trabecular space were observed in AKG supplemented group. The trabeculae in bone metaphysis were also significantly thicker after AKG supplementation. AKG promoted fibrillogenesis in articular cartilage, as indicated by increased cartilage oligomeric matrix protein immunoexpression. By improving the structure and maintaining the proper bone turnover rate of highly reactive and metabolically active medullar and trabecular bones AKG showed its anti-osteoporotic action in laying hens.

## 1. Introduction

About 30% of laying hens, artificially selected for increased egg output, suffer excessive bone loss and fracture, which are mainly detected after slaughter [1]. Bone weakness and fragility, ultimately resulting in bone fractures in laying hens, generally results from decreased bone mass, a pathological condition observed throughout lay, which causes economic losses and negatively affects bird welfare [2,3,4]. Problems with regards to the weight-carrying capacity of the hens’ legs, collapse of the vertebrae and paralysis are also commonly observed in laying hens [2,4]. The aforementioned abnormalities are consequences of the increased calcium mobilization for the formation of the eggshell. The incidence of bone loss and fractures is determined by genetics and strain of hen, the housing system, methods of depopulation and nutrition [5,6,7]. Bone breakage due to osteoporosis during processing reduces the marketability of spent laying hens and increases the need to develop humane on-farm killing methods to support alternative ways of disposing of the spent hens [8]. Thus, proper bone metabolism is an important aspect of the welfare of laying hens [9,10].

For nearly over two decades the effects of alpha-ketoglutarate (AKG), also known as 2-oxoglutaric acid (2-Ox), a derivative of glutamate, on bone metabolism and intestine development have been studied in different species [11,12,13,14]. AKG is a precursor of glutamine (which is considered a non-essential amino acid), the nutritive protective role of which has been highlighted in numerous in vitro studies in both human and animal models, including in birds such as the turkey, hen or broiler chickens [15,16,17]. Glutamine serves as a precursor for ureagenesis, gluconeogenesis and acute-phase protein synthesis, and plays an important role in the inter-organ flow of nitrogen and carbon [18]. AKG serves as a significant energy source for cells. It stimulates the synthesis of proline and hydroxyproline, which are primary substrates for bone collagen synthesis, and enhances bone tissue formation, which is linked with higher serum concentrations of bone alkaline phosphatase activity. It increases protein synthesis and inhibits protein degradation in the liver [19,20,21]. AKG is also a precursor of arginine and other active compounds that are important in the regulation of protein metabolism in skeletal muscle [17,19]. Dietary AKG is absorbed in sufficient quantities from the gastrointestinal tract [22] into the portal circulation [23] to provide a useful resource to increase glutamate concentrations in extra-intestinal tissues [19]. Thus, AKG is widely used in animals and humans as a feed additive [17,24,25,26,27]. In the animal production industry, AKG effectively improves growth performance, nitrogen utilization, immunity, bone development, intestinal mucosal injury, as well as the oxidative system [28].

There is a dearth of information regarding the long-term effects of long-lasting treatment with AKG on bone and cartilage tissue in laying hens. Previous studies have shown that performance parameters of broiler chickens were optimized following 1% glutamine supplementation [29,30]. Dietary AKG supplementation at a concentration of 1% has also been shown to alleviate mucosal damage and increase absorptive function [31,32]. Moreover, there is no data concerning the meat quality in AKG supplemented birds, and due to the fact that meat hens is used for broth in traditional old Polish cuisine [33], there is a need for the assessment of cholesterol or basal physical properties of meat.

Therefore, in the present study, we tested the hypothesis that AKG, through its multiple direct and indirect actions on protein metabolism, could be a valuable supplement not only in the improvement of cartilage and bone quality in laying hens, but also enteroplasticity, and general metabolism.

Our choice of the research objectives aimed at achievement thereof includes bone mechanical testing and dual X-ray absorptiometry, serum hormonal analysis, application of light microscopy, in combination with histochemical and immunohistochemical methods in bone, cartilage and intestine analysis, and comprehensive analysis of muscle quality (muscle fatty acids profile, technological properties and microstructure of breast muscles) in laying hens supplemented with 1% of AKG during the laying period between the 31st–60th week of age.

## 2. Materials and Methods

### 2.1. Ethical Approval

All the chickens were housed in the chicken facility of the Department of Animal Nutrition and Feed Science, National Research Institute of Animal Production, Balice, Poland, which was built in accordance with the Polish Animal Welfare Act (Legislative Decree 2139/2016) and related laws. The experiment was carried out in compliance with the European Union law (Directive 2010/63/UE, received in Poland by Legislative Decree 266/2015) of the European Parliament and of the Council on the protection of animals used for scientific purposes. All of the Polish authors held the certificates for experimentation on living animals delivered by the Polish Veterinary Services, as required by EU law. According to the EU/PL law, approval from an animal ethics committee was not required, as the chickens were fed a non-toxic diet [34], the added feed additive is approved by the EFSA for poultry [15,24], and the birds were not subjected to any invasive procedures. Polish regulations allow for this type of trial to be run on livestock animals without particular per case agreement by the Ethical Committee, as long as no procedures are performed that might cause suffering, pain or other stress, as was the case with the current study. Throughout the experimental period, birds were regularly monitored by a veterinarian.

### 2.2. Birds and Experimental Diets

The experiment was carried out using 48, 17-week-old, Bovans Brown hens, obtained from a commercial source. Hens were housed in a poultry house, in cages (two birds per cage), on a wire-mesh floor, under controlled climate conditions (temperature 19 °C +/− 2 °C; 50–65% of relative humidity; 10 lux of light intensity). The cage dimensions were 30 × 120 × 50 cm, equating to 3600 cm^2^ of total floor space. During the pre-experimental period, lasting from 18th to 30th week of age, all hens were fed the same pre-laying standard, commercial diet (18–20 wks, Supplementary Table S1) and laying diet (21–30 wks), the same as during the experimental period (Table 1). The experimental period ran from the 31st to the 60th week of age of the birds. During the experimental period, the hens had free access to standard commercial laying hens’ mash feed and water, and were exposed to a 14 L:10 D lighting schedule. The composition of the standard commercial (basal) diet is given in Table 1. The composition and nutrient levels of all diets met the nutrient specifications according to the Polish-recommended requirements [34]. The calculated amino acid content in the experimental basal diet is given in Supplementary Table S2. At the start of the experimental period, hens were randomly assigned to one of the two experimental groups: the control group (the CONT group) or the group supplemented (in the diet) with AKG (the AKG group), each compromising of 12 replicate cages with two birds per cage. The control group was fed the basal diet, the AKG group was fed the basal diet plus 1.0% AKG (α-Ketoglutaric acid, #75890, Sigma-Aldrich, St. Louis, MO, USA), which was chosen based on the literature, where the selected criterion was the most optimal performance effect in broiler chickens and laying hens [15,29,30,31,32,35]. The glutamine concentration in the basal diet was not determined. During the experiment, feed intake and laying rate were recorded. At the end of the experiment, one hen from each replicate cage was randomly selected (n = 12 in each group) and slaughtered. Hens were weighed following euthanasia. Blood was collected post-mortem from all slaughtered birds, and the serum was separated by centrifugation at 3000 rpm for 15 min at 4 °C, and stored at −20 °C until further analysis. Samples of the small intestine and breast muscle (*Pectoralis major*) of each bird were collected, and stored until further analysis.

### 2.3. Serum Biochemical Analysis

The blood serum concentrations of insulin-like growth factor 1 (IGF-1), osteoprotegerin (OPG), growth hormone (GH), insulin, bone alkaline phosphatase (BALP), ghrelin, interleukin-1β (IL-1β), leptin, osteocalcin (OC), receptor activator of nuclear factor-kappa Β ligand (RANKL), tumor necrosis factor alpha (TNF-α) and vitamin D (VitD, general 1,25-dihydroxyvitamin D3, calcitriol) were determined using commercial, chicken-specific, enzyme-linked immunosorbent assay (ELISA) kits: IGF-1 (E-EL-Ch0116; Elabscience Biotechnology Co., Ltd., Wuhan, China), OPG (E-EL-Ch1455; Elabscience Biotechnology Co., Ltd., Wuhan, China), GH (E-EL-Ch2116, Elabscience Biotechnology Co., Ltd., Wuhan, China), insulin (CSB-E13293C, Cusabio Technology LLC., Wuhan, China), BALP (E-EL-Ch0894, Elabscience Biotechnology Co., Ltd., Wuhan, China), ghrelin (abx190771, Abbexa Ltd., Cambridge, UK), IL-1β (E-EL-Ch0539, Elabscience Biotechnology Co., Ltd., Wuhan, China), leptin (abx150041, Abbexa Ltd., Cambridge, UK), OC (E0244Ch, Bioassay Technology Laboratory, Shanghai, China), RANKL (E0308Ch, Bioassay Technology Laboratory, Shanghai, China), TNF-α (E0025Ch, Bioassay Technology Laboratory, Shanghai, China), VitD (RK00649, Abclonal Biotechnology, Woburn, MA, USA). All procedures were performed according to the manufacturer’s protocols. Samples (n = 12 in each group) were analyzed in duplicate using a Benchmark Plus microplate spectrophotometer (Bio-Rad Laboratories, Inc., Hercules, CA, USA). Results were calculated using standard curves created in individual tests; the coefficient of determination of the standard curves was above 0.980.

Analyses of blood serum total cholesterol were performed colorimetrically on a Mindray BS-120 apparatus (Bio-Medical Electronics, Shenzhen, China), using a commercial kit (Alpha Diagnostics, Warsaw, Poland).

### 2.4. Intestine Tissue Histomorphometric Analysis

Two, 10 mm long segments of the small intestine, from the duodenum (2 cm distal to the pylorus) and 50% of the total intestinal length were collected from each bird immediately after slaughter. The tissues were fixed in phosphate-buffered, 4% paraformaldehyde (pH 7.0), dehydrated through a graded ethanol series, fixed with nonpolar Ottix Plus and Ottix Sharper solvents (DiaPath, Martinengo, Italy) and embedded in paraffin. Twenty cros- sections (with 20 μm interval after every 6 slices) of 5 μm thick were cut with an HM 325 microtome (Thermo Fisher Scientific, Waltham, MA, USA) and stained with Masson’s trichrome [37]. Microscopic images were collected using a CX43 microscope (Olympus, Tokyo, Japan). The small intestine structure was examined using Olympus cellSens software (Olympus, Tokyo, Japan) and ImageJ [38]. The following morphometric variables of the intestine were analyzed: villus thickness and length, crypt width and depth, villus length to crypt depth ratio and small intestine absorptive surface, as described by Kisielinski et al. [39].

### 2.5. Analysis of Meat Microstructure, Physical Properties, Fatty Acid Profile and Cholesterol Content

For microstructural analysis, samples of the left breast muscle (*Pectoralis major*) of each bird were taken within 15 min after slaughter. Muscle samples were cut into 1 cm^3^ pieces (parallel to the muscle fibers) and frozen in isopentane that was cooled using liquid nitrogen and stored at −80 °C until subsequent analyses. Samples were mounted on a cryostat chuck with a few drops of Tissue-Tek tissue-freezing medium (Sakura Finetek Europe, Zoeterwoude, The Netherlands). Transverse sections (10-µm thick) were cut at −20 °C in an MEV cryostat (Slee Medical, Mainz, Germany) and stained using haematoxylin and eosin. A minimum of 100 fibers were counted in each section using a NIKON E600 (Nikon, Tokyo, Japan) light microscope. The muscle fiber diameter was quantified with an image analysis system using Multi Scan v. 14.02 (Computer Scanning Systems Ltd., Poland) [40].

Samples of the right breast muscle of each bird were used for the assessment of meat quality. The initial (pH_15min_) and final pH (pH_24h_) of the breast muscle were determined at 15 min and 24 h post-mortem, respectively, using a pH-Star pH meter (Matthäus, Pöttmes, Germany). The measurement was made by placing the electrode at an angle of 45°, halfway through the total thickness of the breast muscle.

The meat colorimetric measurements were assessed 15 min post-mortem using a CR-221 chroma meter (Minolta, Osaka, Japan) with the diameter of estimated measuring area of 3 mm on the inner side of the breast muscle, immediately after they had been detached from the bone. The entire area of the analyzed muscles was measured, with four measurements per muscle (in the whole part, from the cranial to caudal part) and a calculation of the mean for individual color parameters. CIE Lab color scale (L*—lightness, a*—redness, and b*—yellowness) specified by the International Commission on Illumination (Commission Internationale de l’Éclairage, CIE) [41] was used; hue angle and chroma were also calculated [42].

Drip loss was determined from meat weight loss after 24 h of cold storage at 4 °C. To this end, samples weighing 50 g were collected from the cranial part of breast muscle 15 min *post-mortem* and placed in airtight containers. The drip loss was determined as a percentage loss of initial weight [43].

Meat samples for Warner-Bratzler shear force (WB) were taken after 24 h of cooling at 4 °C. Breast muscle fragments (from cranial part of *Pectoralis major*) weighing ca. 90 g were cooked in individual plastic bags in a water bath at 100 °C, until a core temperature of 76 °C was obtained in the thickest part of the sample. Next, the samples were chilled at room temperature for 30 min and kept in cold storage at 4 °C for 45 min. A core (1.27 cm in diameter and ~3 cm in length) was cut from the thickest portion of each cooled sample, parallel to the fiber orientation. The shear force was determined by cutting obtained samples perpendicular to the direction of the muscle fiber, using an INSTRON 5542 system (Instron, High Wycombe, England) equipped with a Warner-Bratzler blade.

Analysis of the fatty acid profile of the meat was performed according to the methods described in Domaradzki et al. [44]. Brieflyfatty acid methyl esters (FAMEs) were analyzed chromatographically (CG 3900 gas chromatograph; Varian, Walnut Creek, USA) following fat extraction from freeze-dried meat samples. FAMEs were separated in a CP 7420 capillary column (Agilent Technologies, Santa Clara, CA, USA). Partial sums, related indices and ratios of fatty acids were calculated according to the following formulas: long-chain n-6 fatty acids (LC n-6: sum of 20:2 n-6, 20:3 n-6, 20:4 n-6, 22:4 n-6 and 22:5 n-6); long-chain n-3 fatty acids (LC n-3: sum of 20:3 n-3, 20:4 n-3, 20:5 n-3, 22:5 n-3 and 22:6 n-3); long-chain polyunsaturated fatty acids (LC-PUFAs: sum of LC n-6 and LC n-3); conjugated linoleic acid (ΣCLA: sum of 18:2 c9,t11, 18:2 t9,c11, 18:2 t7,c9, 18:2 t11,c13, 18:2 t12,c14, 18:2 t11,t13; ΣC18:2 trans): sum of non-conjugated (18:2 t,c/ c,t/ t,t isomers; ΣTFA: sum of ΣMUFA trans and ΣC18:2 trans) [44].

The cholesterol content in the breast muscles (samples from the right breast muscle obtained after 24 h colling at 4 °C and frozen at −20 °C until analysis) was analyzed colorimetrically using the method described by Rudel and Morris [45]. A meat sample (1 g) was homogenized with 3 mL 95% ethanol and 2 mL 50% potassium hydroxide. The homogenates were incubated in a water bath at 60 °C for 10 min and then allowed to cool to room temperature. Hexane (5 mL) was then added to the homogenates and mixed for 20 s. Deionized water (3 mL) was then added to the final homogenate and vortexed and then allowed to settle at room temperature for 15 min to complete phase separation. About 2.5 mL of the upper phase (hexane layer) was transferred into a clean glass tube followed by evaporating the hexane to dryness under nitrogen gas flow at 60 °C. The residue was re-suspended with 4 mL o-phthalaldehyde reagent and kept at room temperature for 10 min, after which 2 mL of concentrated sulphuric acid was slowly added, mixed and allowed to stand for an additional 10 min at room temperature [46]. The concentration of cholesterol in the prepared samples and cholesterol standards (L-4646, Sigma-Aldrich, St. Louis, MO, USA) was measured at an absorbance of 550 nm, using a Unicam Helios alpha spectrophotometer (Thermo Spectronic, Cambridge, UK). Cholesterol content was expressed in mg in 100 g of meat.

### 2.6. Bone and Cartilage Analysis

Immediately after euthanasia, the left tibia from each bird was carefully dissected out and then cleaned of adhering tissues and frozen at −20 °C. After thawing overnight at 7 °C the tibia was weighed and measured for length, after which the Seedor index (bone weight to length ratio, which can be used to describe the degree of bone mineralization) and relative bone weight (bone weight to body weight ratio) were calculated. The measurement of whole-bone mineral density (BMD) was performed using the dual-energy X-ray absorptiometry (DXA) method on a Lunar densitometer (GE, Madison, WI, USA). The three-point bending test was performed on a Zwick Z010 universal testing machine (Zwick-Roell GmbH and Co., Ulm, Germany) [46]. A fixed span of 50 mm (about 40% of total bone length) and a constant load rate of 10 mm/min were used. Next, the tibia was cut across, at the midpoint of the bone diaphysis, with an MBS 240/E diamond bandsaw (Proxxon GmbH, Foehren, Germany) and bone diaphysis geometric parameters (cortical index, cross-sectional area, mean relative wall-thickness, and cross-sectional moment of inertia) were determined on the basis of measurements of cortical bone cross-sectional diameters using a digital caliper [46]. Finally, on the basis of recorded load-deformation curves and measured diameters, bone mechanical (yield load, fracture load, elastic work, work to fracture, and stiffness) and material properties (Young’s modulus, yield strain, fracture strain, yield stress, and fracture stress) were determined using standard engineering beam-theory equations, using Origin software (OriginLab, Northampton, MA, USA) [45,47].

Samples of the lateral condyle (5 mm thick) were cut perpendicularly to the articular surface using an MBS 240/E diamond bandsaw. Similarly, 5 mm thick samples of the bone diaphysis, containing medullary bone, were cut at the midpoint of the bone diaphysis. Samples were fixed in phosphate-buffered 4% paraformaldehyde (pH 7.0), decalcified in a 10% EDTA solution (pH 7.4), dehydrated through a graded ethanol series, fixed with nonpolar Ottix Plus and Ottix Sharper solvents (DiaPath, Martinengo, Italy), embedded in paraffin, cut with a microtome at a thickness of 4 μm. Slides were stained with Picrosirus red (PSR) stain in order to evaluate basal morphology, and the distribution of immature (thin, green) and mature (thick, red) collagen fibers [48]. Stained slides were observed in brightfield (histomorphometry) and polarized (collagen distribution) light using a light microscope (CX43, Olympus, Tokyo, Japan). ImageJ software was used to assess the morphology of trabecular bone, the relative bone volume (BV/TV), mean and max trabecular thickness (Tb.Th), mean and max trabecular separation (Tb.Sp), and trabecular number (Tb.N) from the microscopic images of the bone trabeculae and medullary bone [26,49].

Immunohistochemical staining of articular cartilage sections was performed according to the protocols provided by the producer of the antibody. Briefly, slides were deparaffinized in xylene and rehydrated, endogenous peroxidase activity was then blocked with 3% H_2_O_2_ (Sigma-Aldrich, St. Louis, MO, USA). The enzymatic antigen retrieval step was performed by 10-min incubation with proteinase K (Sigma-Aldrich, St. Louis, MO, USA) at 37 °C. Primary antibody targeting cartilage oligomeric matrix protein—COMP (Elabscience Biotechnology Co., Ltd., Wuhan, China, dilution 1:100 in Diamond antibody diluent, Cell Marque Corp., Rocklin, CA, USA)—was incubated at room temperature for 1 h. Ready-to-use Bright Vision +Poly-HRP-Anti Ms/Rb IgG Biotin-free (Immunologic, Duiven, Netherlands) served as the secondary antibody. DAB (Abcam, Cambridge, UK) was used as a substrate-staining chromogen. Slides were counterstained with hematoxylin (Sigma-Aldrich, St. Louis, MO, USA) and mounted with DPX mounting medium (Sigma-Aldrich, St. Louis, MO, USA). Slides were examined using a CX43 microscope (Olympus, Tokyo, Japan).

### 2.7. Statistical Analysis

All statistical procedures were conducted using Statistica software (v. 13.0, TIBCO Software Inc., Palo Alto, CA, USA). A cage was considered as the experimental unit for daily feed intake and laying rate (n = 12 per group), whereas for all other traits a hen was considered the experimental unit (n = 12 per group). This sample size was calculated for a two-sided t-test of two groups with an α of 0.05 and power at 0.8, with an effect size of 1.2 [50]. Available literature data on laying hens show that for these assumptions n = 12 sample size has a power of 80% to detect a change of 5% in tibia breaking strength [51], a change of 8% in blood serum cholesterol content [52], and a change of 3% and 7% in duodenum villus length and crypt depth, respectively [53], assuming a 5% significance level. The normality of the data was tested using the Shapiro-Wilk normality test and the homogeneity of variances was tested using Levene’s test. Normally-distributed variables were analyzed using a two-tailed Student’s t-test; non-parametric data or data with non-homogeneous variances were analyzed using a Mann-Whitney U test. For all tests, a *p*-value < 0.05 and a confidence interval of 95% were established. Effect sizes (ES) for all significant comparisons were estimated with Cohen’s d for parametric comparisons and Cohen’s r for non-parametric comparisons [54].

## 3. Results

### 3.1. Performance

Daily feed intake, bodyweight of laying hens (at the beginning and the end of the experiment) and their laying rates were not different between groups (Table 2).

### 3.2. Blood Serum Biochemical Parameters

Dietary supplementation with AKG resulted in an increase in serum bone alkaline phosphatase (188%, ES = 1.19), RANKL (72%, ES = 0.36), leptin (20.7%, ES = 0.85) and ghrelin (35%, ES = 2.08), and a decrease in OPG (13.1%, ES = 0.96) and OC (21.4%, ES = 1.15). Moreover, the concentration of total cholesterol decreased by 8.1% (ES = 1.15). No other significant changes in serum biochemical parameters were observed (Table 3).

### 3.3. Intestinal Histomorphometry

**Figure 1:**
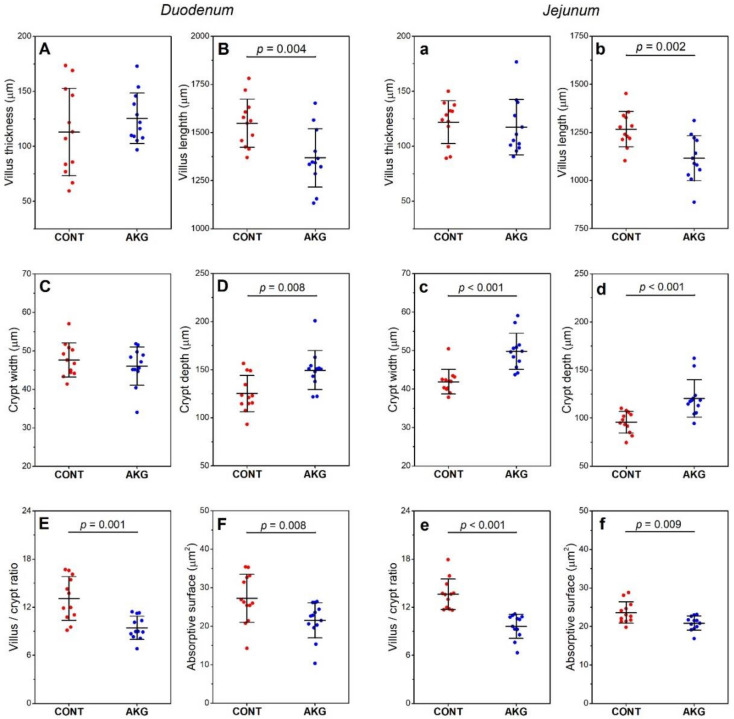
The effect of dietary supplementation with AKG during the experimental period (31–60 weeks of age) on the intestinal histomorphometry of laying hens at the age of 60 weeks in the duodenum (**A**–**F**) and jejunum (**a**–**f**). The control (CONT) group included laying hens not supplemented with AKG; the AKG group included laying hens supplemented with AKG at a dose of 1.0% (10 g/kg feed). Data are mean values ± standard deviation (whiskers) from n = 12 laying hens.

A decrease in villus length (11.6%, ES = 1.24, Figure 1B) and an increase in crypt depth (19.2%, ES = 0.76, Figure 1D) was observed in the duodenum after AKG supplementation, which in turn lead to a decrease in duodenal villus/crypt ratio (28.2%, ES = 0.92, Figure 1E) and absorptive surface (20.8%, ES = 0.76, Figure 1F). In the jejunum, a decrease in villus length (11.9%, ES = 1,37, Figure 1b) and an increase in crypt width (18.8%, ES = 1.08, Figure 1c) and depth (26.3%, ES = 0.97, Figure 1d) were observed following AKG supplementation, which lead to a decrease in the villus/crypt ratio (29.4%, ES = 2.18, Figure 1e) and absorptive surface (11.4%, ES = 1.10, Figure 1f).

### 3.4. Breast Muscle Microstructure, Physical Properties, Fatty Acid Profile and Cholesterol Content

Both the absolute and relative breast muscle weights, as well as the other physical properties of the breast muscles, were similar in both the control and AKG supplemented groups (Table 4). A comparison of the analyzed breast muscles revealed a significantly higher shear force (27.4%, ES = 1.57), drip loss (34%, ES = 1.12) and a significantly decreased hue angle (26.4%, ES = 0.73) and cholesterol content (29.75%, ES = 1.20) following AKG supplementation.

No significant changes in skeletal muscle fatty acid composition were observed following AKG supplementation (Table 5).

### 3.5. Bone and Cartilage Analysis

**Figure 2:**
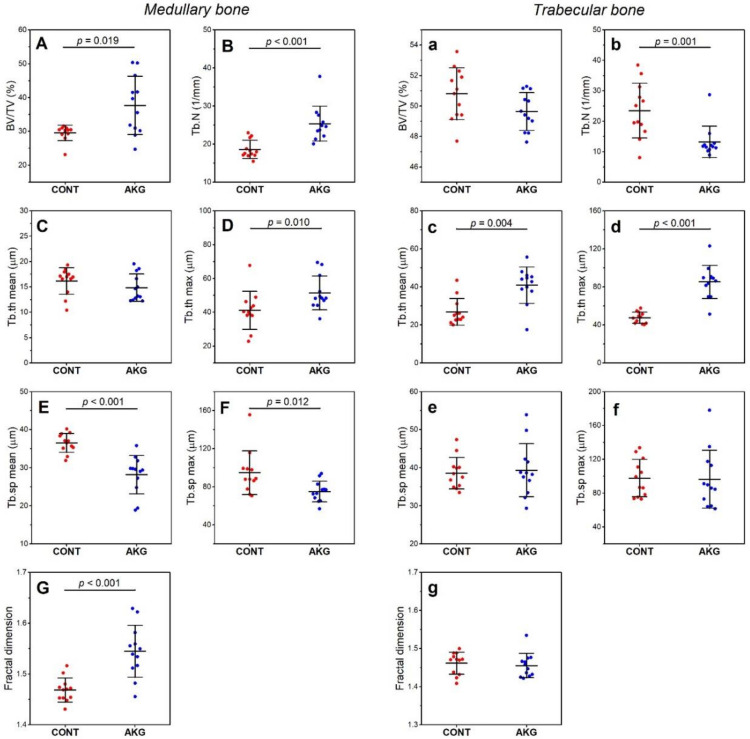
The effect of dietary supplementation with AKG during the experimental period (31–60 weeks of age) on the histomorphometry of tibial medullary bone (**A**–**G**) and trabecular bone (**a**–**g**) in laying hens at the age of 60 weeks. BV/TV—relative bone volume; Tb.N—trabecular number; Tb.Th—trabecular thickness; Tb.Sp—trabecular separation. The CONT group included laying hens not supplemented with AKG; the AKG group included laying hens supplemented with AKG at a dose of 1.0% (10 g/kg feed). Data are mean values ± standard deviation (whiskers) from n = 12 laying hens.

General, geometric and mechanical (structural and material) properties of the tibia are presented in Table 6, while basal histomorphometrical parameters of medullary and trabecular bone of the tibia are presented in Figure 2. Supplementation with AKG did not influence general, geometric and mechanical (structural and material) properties of the tibia.

Histomorphometrical analysis indicated that AKG increased medullary bone volume (BV/TV, by 27%, ES = 0.67, Figure 2A and Tb.N, by 36%, ES = 1.06, Figure 2B), resulting in an increase in maximal trabecular thickness (24.9%, ES = 0.76, Figure 2D) and a decrease in mean (22.8%, ES = 2.00, Figure 2E) and maximal (20.9%, ES = 1.06, Figure 2F) trabecular space. Fractal dimension increased in medullary bone by 5.2% after AKG supplementation (ES = 1.84, Figure 2G). The number of trabeculae in cancellous bone decreased (by 43.9%, ES = 0.94, Figure 2b) and mean and maximal trabecular thickness increased by 52.2% (ES = 0.83, Figure 2c) and 79% (ES = 0.99, Figure 2d), respectively, following AKG supplementation. No other significant differences in trabecular bone were noted.

**Figure 3:**
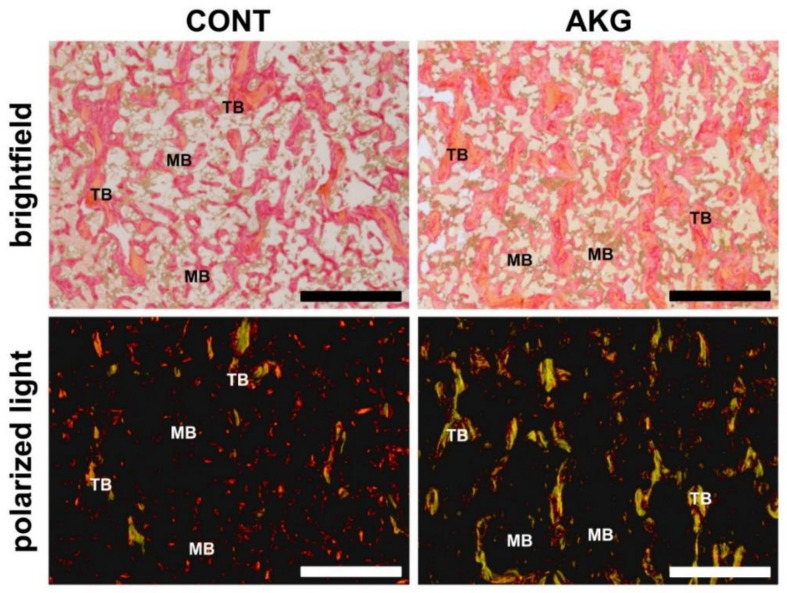
The influence of AKG supplementation during the experimental period (31–60 weeks of age) on bone collagen in laying hens at the age of 60 weeks. Representative images of PSR-stained sections from the tibial trabecular (TB) and medullary (MB) bone of laying hens from the control (CONT) and AKG supplemented (AKG) groups, viewed with conventional brightfield light microscopy and polarized (polarizer and analyzer are “crossed”) light. The large mature collagen fibers are orange or red and the thick ones, including the reticular fibers, are green. All scale bars represent 100 μm.

**Figure 4:**
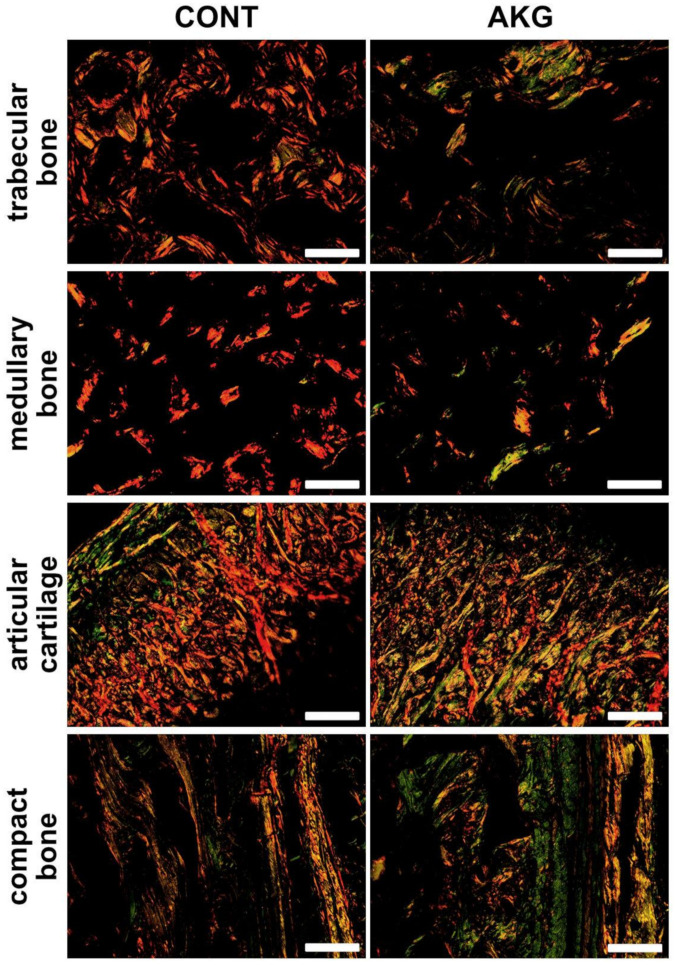
The influence of AKG supplementation during the experimental period (31–60 weeks of age) on bone collagen in laying hens at the age of 60 weeks. Representative images of PSR stained sections carried out on formaldehyde-fixed sections from the tibial articular cartilage and bone of laying hens from the control (CONT) and AKG supplemented (AKG) groups, observed in polarized light. The large mature collagen fibers are orange or red and the thick ones, including the reticular fibers, are green. All scale bars represent 100 μm.

Structural information obtained from the analysis of collagen fibers in PRS stained sections showed both type of fibers with the predominance of mature, thick (red) collagen in the articular cartilage, cancellous, compact and medullary bone in the control group of laying hens, while immature, thin (green) collagen fibers were dominant in all types of bone and articular cartilage in the hens supplemented with AKG (Figure 3 and Figure 4).

**Figure 5:**
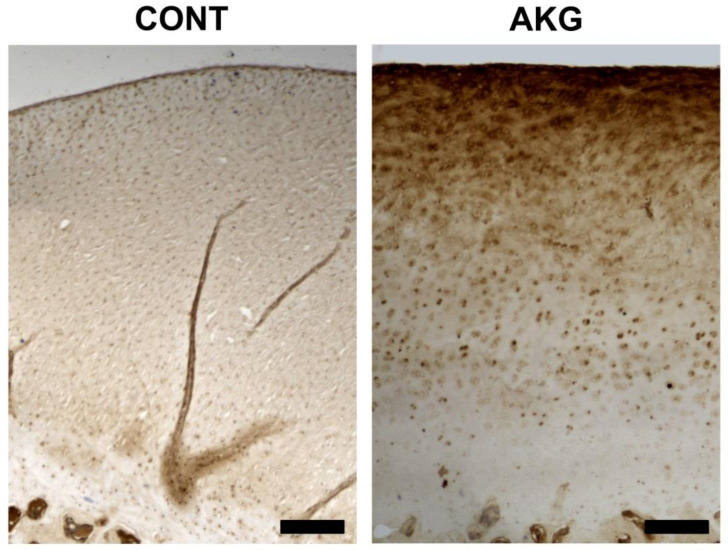
The influence of AKG supplementation during the experimental period (31–60 weeks of age) on the distribution of cartilage oligomeric matrix protein (COMP) in laying hens at the age of 60 weeks. Representative pictures of the immunohistochemical analysis of COMP carried out on formaldehyde-fixed sections from the tibial articular cartilage of laying hens from the control (CONT) and AKG supplemented (AKG) groups. Cartilage from the control and AKG supplemented hens show differences in the quantity and distribution of COMP (brown color). Tissues with anti-COMP antibody, control cartilage, show a lower accumulation of COMP throughout the whole thickness of the cartilage, while cartilage from the AKG-supplemented hens shows denser and darker staining, indicating higher COMP deposition, especially in the upper zones of the cartilage. All scale bars represent 200 μm.

The analysis of COMP immunostaining in the articular cartilage showed differences in the quantity and distribution of COMP (Figure 5; brown color). Tissues with anti-COMP antibody, control cartilage showed lower accumulation of COMP through the whole thickness of the cartilage, while cartilage from the AKG-supplemented hens showed denser and darker brown staining, indicating higher COMP deposition, especially in the upper zones of the cartilage.

## 4. Discussion

Dietary supplementation with AKG, at a concentration of 1%, had no significant effects on the feed intake and body weight of laying hens in the current study. Even though our experiment lasted for 30 weeks, similar results were obtained in a previous study in which hens were supplemented with 1% glutamine for a period of 56 days and feed intake was unaffected [35]. Dietary glutamine supplementation has also been shown to have no effect on the feed intake [55,56] or bodyweight [29,57] of broiler chickens. However, one previous study showed that broiler chickens fed a diet supplemented with 1% glutamine weighed significantly more compared to those in the control group and those in groups fed diets supplemented with 0.5, 1.5 or 2% glutamine. Moreover, this study proves that higher concentrations of dietary glutamine could be toxic to broiler chickens [29]. The literature also reveals that the range of doses of glutamine that result in positive effects with regards to zootechnical performance is rather small; supplementation above 1% predisposes to a reduction in body weight [29,58]. Other studies in which turkeys were supplemented with AKG at a dose of 0.4g/kg b.w. have also observed no effect on body weight [20,22]. These data suggest that glutamine or derivatives of glutamine do not influence body weight, irrespective of the age of birds (growing broilers or adult hens). These findings are however in contrast to the data obtained from studies performed on mammals [59,60].

Improvements in growth and performance parameters, as well as better bone metabolism, are dependent on intestinal morphology and physiology. Villus length and crypt width determine the intestinal absorptive surface and nutrient absorption. A surface area of the small intestine is a key determinant of the absorptive capacity [61]. Moreover, in the assessment of the intestinal response to various diets, the determination of the villus to crypt ratio is also effective [62]. Glutamine, due to it being the most abundant amino acid in blood plasma, is the main source of metabolic fuel for enterocytes and exerts trophic effects (including crypt cells) on the intestinal mucosa. Glutamine promotes the growth and proliferation of the intestinal mucosa as well as improves epithelial integrity [63,64]. In a study performed on young mammals fed a diet enriched with 0.5% glutamine, longer villi and deeper crypts, with a reduced villus/crypt ratio were observed [65]. The effects on duodenal villi length seen after glutamine (1%) administration are not reproduced with the application of other amino acids [30,58]. Broiler chickens fed diets supplemented with glutamine at a concentration of 1% (10 g/kg of feed) have longer villi, deeper crypts and a higher villus/crypt ratio in each part of the intestine, compared to those fed a glutamine free diet [29,30,57,66]. Contrary to results obtained in broiler chickens, glutamine supplementation to laying hens at the same concentration of 1% has been shown to have no effect on the morphometry of the duodenum, jejunum and ileum [35]. Our presented results were quite different. Dietary supplementation with AKG, a derivative of glutamine, resulted in decreased crypt depth in both the investigated segments of the intestine, and lead to a decrease in crypt width in the jejunum in our laying hens. Probably, there was one underlying mechanism by which villi became shorter when more glutamine is delivered to mucosal cells. The growth of a villus is an effect of decreased exfoliation of apoptotic cells at the top of the villi, and not increased mitotic activity in the zone of renewal in the intestinal crypts [67]. Probably, when more glutamine is delivered, enterocytes move faster from the renewal zone towards the top of the villus due to intensified proliferation [63]. The epithelial cells then mature, differentiate rapidly and undergo apoptosis, leading to villus shortening. However, this result indicates the need of further studies.

Furthermore, this discrepancy between our presented results with regards to previous studies showing improved intestinal morphology in AKG supplemented broiler chickens could be due to the fact that broiler are still growing birds, whereas our hens were adult. However, in both cases, in growing broiler chickens and in hens within the production cycle, the intestinal tract is short and intestinal requirements for energy and nutrients are very high. Feed intake and bodyweight, as well as laying rate, were similar in both groups in our study. It indicates that nutrients utilization in AKG supplemented hens could be more effective, which is supported by improved bone parameters. Other mechanisms through which glutamine and glutamine derivatives may exert their effects on the functioning of living organisms include their influence on the endocrine system, since AKG is converted into glutamine and glutamate and then into, arginine [17], which in turn stimulates the secretion of growth hormone (GH), insulin and IGF-I [19].

In the current study, dietary AKG supplementation had no significant effects on serum insulin, growth hormone or IGF-I concentrations in laying hens. These results are contrary to those of previous studies in which AKG supplementation to growth-retarded mammals enhanced body weight, while simultaneously increasing growth hormone, IGF-1, as well as BALP concentrations [60,68]. Growth hormone, a pleiotropic hormone that plays an important role in the modulation of various physiological processes, is not a good indicator of growth in adults, although it positively correlates with the conversion of dietary protein to body protein. Additionally, in birds, growth hormone plays a special role in short-term metabolism. For this reason, plasma concentrations of growth hormone decline with age earlier in broiler chickens than in laying birds [69]. Growth hormone exerts anabolic effects through the stimulation of IGF-1 production, however, it can also act through IGF-1-independent pathways [63]. In post-hatching development, IGF-1 activity is maximal in the early stages of life when maximal growth in avian species is observed. Growth hormone plays a very important physiological role in the hen’s ovulatory cycle, in relation to the metabolic demands of yolk synthesis and deposition [69,70].

In the current study, both groups of laying hens had the same laying rate, irrespective of treatment. However, the aim of the study was not to assess the quality of the eggs. Thus, both egg and eggshell quality should be investigated in further studies.

As mentioned above, no differences in serum insulin concentrations were observed in the current study between the control and AKG supplemented groups. Insulin has multiple functions within insulin receptor-expressing tissues, including in the hypothalamus, adipose tissue, muscle, and liver. Peripheral insulin serves as a peripheral signal of the energy state of birds, stimulates protein synthesis and suppresses protein degradation [71]. Feed consumption in layer birds is significantly inhibited under both fasting and ad libitum conditions in the case of an increase in peripheral insulin [72]. On the other hand, insulin resistance exists in the central nervous system of broiler chickens (in mammals it acts as a catabolic hormone in the central nervous system), possibly due to persistent hyperinsulinemia, which results in a down-regulation of insulin receptor expression in the central nervous system compared to that observed in layer birds. Differences in energy metabolism exist between chickens and layers. Broiler chickens consume more feed than layers and have increased the bodyweight gain and clearance of triacylglycerides, as well as lower protein degradation and basal metabolic rate, due to a genetically changed control mechanism of feed intake [16]. Hyperinsulinism in broiler chickens causes excessive elevation of blood lipids [73]. Laying hens have to meet the high demands of lipid synthesis and transport due to egg formation. Insulin controls the level of liver lipogenic enzymes. Lipid metabolism and synthesis in laying hens occurs much faster than that in broilers and mammals, and differs substantially from that known in mammals [74]. Insulin signaling in the chicken liver is similar to that in mammals, however, insulin signaling in the muscles of chickens is unlike that which occurs in mammals [75].

Broiler muscle has been well characterized as a result of the numerous studies that have been performed in order to determine the role of genes, environmental factors and nutrition on the quality of broiler meat [33,76,77]. On the other hand, studies analyzing the meat quality of laying hens are rare and this type of research is needed because their carcasses could be attractive as a raw material for consumption. A previous study performed on four different lines of laying hens showed that the cholesterol content of breast muscle depends on the line of hen [33,77]. In the current study, only one line of the hen was used, in which a decrease in total cholesterol content of the breast muscle was observed (for the first time) after AKG supplementation. It should be highlighted that the concentration of total cholesterol in the serum of our laying hens was also decreased after AKG supplementation. Earlier studies that made use of AKG supplementation in mammals have proven the beneficial effects of glutamine derivates on serum cholesterol [13,78]. From the point of view of the consumers, this decrease in cholesterol content in the meat of hens is favorable. Further studies should be performed to assess whether the same effect is observed in the egg yolk.

There is a scarcity of data concerning the fatty acid profile of skeletal muscle following AKG supplementation to laying hens. Analysis of the breast muscle fatty acid profile in the current study indicated no significant effects of dietary AKG in the hens at 60 weeks of age. However, it is unknown whether the same results would be observed with regards to the fatty acid composition of the egg yolk lipids, since they were not assessed in the current study and thus further studies and analyses are needed. Previous studies have shown that it is possible to alter the fatty acid profile of egg yolk lipids through dietary manipulation, including that of LC PUFA n-3 [79,80].

Meat color is also an important parameter and correlates with other qualitative meat traits and is also equated with freshness. Colour is the easiest determinant of sensory quality. Meat color depends on three physical parameters: dominant wavelength, saturation and lightness, which all depend on myoglobin content [81]. Dietary AKG supplementation in the current study showed no significant effects on breast muscle color in general (L*, a* and b*), however, hue values were lower after AKG supplementation, indicating less red color, which is within consumer expectations. In traditional, old Polish cuisine, meat hens used for broth are expected to be yellow (b*). The comparison of meat obtained from different popular lines of hens meet consumer expectations in this respect [33]. Furthermore, our AKG supplemented hens were characterized by meat with a higher drip loss, despite the lack of changes in lightness. Drip loss corresponds to water-holding capacity, which indicates the ability of meat to hold its own water [33]. Higher drip loss is linked with higher lightness and lower water-holding capacity, which is linked with other parameters relating to the sensory quality of meat, like the juiciness which is dependent on fat content, the tenderness of the meat and the amount of liquid released during grinding. In the current study, water-holding capacity was not measured, however, the higher drip loss observed after the AKG supplementation was probably the result of a lower water holding capacity, which in turn leads to the meat being less juicy. It could be for this reason that a higher shear force was observed, since no increases in fiber diameter of the breast muscles were observed in our AKG supplemented hens. Moreover, with the increasing age of the birds, the tenderness of the meat decreases and the intensity of its palatability increases. In the case of meat obtained from laying hens, the meat characteristics are genetically determined and depend on the type and line of hens, as well as the feeding and rearing conditions as a source of stress [33]. The sensory characteristics of the meat of burrowing poultry are determined at all possible stages of production. AKG supplementation to birds seems to be good practice since it lowers the cholesterol content of the meat.

It should be mentioned once again that feed intake was not different between groups in the current study, despite increases in the serum concentrations of two hormones which are important in regulating food intake, ghrelin and leptin, following AKG supplementation. The mechanism by which ghrelin exerts its effects in the laying hens includes the control of basic ovarian function at the level of both the central nervous system and the gonads. Food consumption affects the production of ghrelin, demonstrating the integration of nutrition and reproduction in laying hens [82]. However, the effect of ghrelin on food or water intake in avian species is opposite to that which is known in mammals and other vertebrate species and involves sleep-like behavior in birds. It has been proposed that different neuroendocrine factors mediate ghrelin-induced changes in feeding behavior in birds as compared to mammals. An increase in peripheral ghrelin also reduces lipogenic-related transcription factors in the liver indicating its important role in lipogenesis in birds [83]. Moreover, ghrelin is found in the proventriculus, duodenum, jejunum, ileum, caecum, and colon but not in the myenteric plexus in the avian species. The number of ghrelin-immunopositive cells increases with age in birds. Ghrelin plays an endocrine role in chickens and stimulates region-specific contraction in the gastrointestinal tract, as well as gastric acid secretion [83].

Leptin is not expressed in the adipose tissue of either broilers or layers, irrespective of the difference in appetite, body growth rate, and reproduction efficiency. Leptin is released mainly by the pituitary gland in birds. Thus, it is clear that adipose tissue in birds does not trigger the signal for fat stores via leptin, which plays an important role in avian energy expenditure. The pattern of leptin expression in birds suggests that it is rather involved in the regulation of reproduction and the stress response, than in the regulation of appetite and bone integrity [84]. A previous study involving heterologous leptin administration to birds showed reduced plasma cholesterol and triglyceride concentrations following the leptin administration [85].

The main effect of leptin and ghrelin in the laying hens supplemented with AKG in the current study was probably the decrease in cholesterol content in the breast meat. More so since no changes in feed intake, body weight or egg production rate was observed following AKG supplementation, despite increased concentrations of both leptin and ghrelin. Moreover, no alterations in three well-known adipokines (TNFα, IGF-1 and IL-6) were observed in the laying hens supplemented with AKG in the current study. These adipokines operate together with leptin in mammals, to control appetite, insulin resistance, and inflammation, and they are expressed at low levels in chicken visceral fat. Serum concentrations of IL-1β were also unchanged after AKG supplementation in the current study, which could be an indication that AKG supplementation did not stimulate the production of these cytokines, thus no local inflammatory response (especially in intestinal mucosa), with the induction of the acute-phase response in the liver (although it was not determined) was observed. Cytokine concentrations were not affected by the AKG supplementation, probably due to the similar physiological status of the hens from both groups, irrespective of the added AKG. Despite similar feed intake, body weight and rate of egg production, the laying hens supplemented with AKG showed decreased serum concentrations of osteocalcin, a major non-collagenous protein in bones, which is synthesized by osteoblasts and involved in bone remodeling in laying hens. In hens, osteocalcin is affected by the sexual maturation process and egg production. Osteocalcin concentration declines after hens start to lay eggs [86]. Moreover, in laying hens additional mechanism of osteocalcin action is observed. The pathophysiological changes associated with fatty liver disorder interfere with the homeostasis of osteocalcin in regulating bone turnover, and thus bone health is reduced [86]. Although in the current study, apart from the cholesterol content of the breast muscle, no other biochemical analyses were performed, the decrease in osteocalcin concentration could indicate that synthesized osteocalcin was incorporated into the bone matrix during bone turnover in producing time in laying hens AKG supplemented. This incorporation was supported by the increase in bone alkaline phosphatase following AKG supplementation in the laying hens.

Bone alkaline phosphatase is a very important marker of bone formation in birds and thus can be detected in the liver, lungs, skeletal muscle, and the heart of birds, contrary to mammals. In avian species, enhanced bone alkaline phosphatase activity relates to increased osteoblastic activity. For this reason, it is used as a marker for the evaluation of skeletal health and bone disease in birds [86].

In addition to growth hormone, IGF-1 and bone alkaline phosphatase, bone and cartilage homeostasis is controlled by the RANK/RANKL/OPG system. Thanks to an appropriate balance between OPG and RANKL, bone formation and bone resorption can exist. Osteoprotegerin (OPG) is a natural inhibitor of the receptor activator of nuclear factor kappa-B ligand (RANKL), a physiologically negative regulator of osteoclastogenesis. It prevents RANKL from binding to RANK, preventing osteoclast production and ultimately leading to bone loss. Thus, osteoclast differentiation is mainly regulated by RANKL. Both OPG and RANKL are expressed by osteoblasts and osteocytes [87].

The analysis of serum concentrations of both OPG, a soluble receptor that binds to RANKL, and RANKL showed that long-term AKG supplementation to hens during the laying period decreased the concentration of OPG and increased RANKL. This could suggest, as in the case of our hens, increased bone remodeling, specifically that of trabecular (structural bone) or medullary bone. Laying hens are in a state of imbalance with regards to bone metabolism during the laying period, which in turn reduces the strength of the bones in laying hens, leading to fractures. However, based on the analysis of mechanical and geometric parameters, it can be stated that the predisposition of tibia bone midshaft to fractures was not affected by AKG supplementation. On the other hand, it is well-known that bone mineralization declines progressively in the structural bones of laying hens throughout the layer period; and that bone remodeling occurs a lot faster in cancellous bone. AKG supplementation to the layer hens in the current study decreased the number of trabeculae in cancellous bone, but they become thinner. Moreover, AKG supplementation was even more effective in medullary bone, where a significant improvement in the bone structure was observed following AKG supplementation. AKG improved the microstructure of medullary trabeculae, which was manifested in raised trabecular thickness and decreased space. Our results are consistent with Fleming, who showed that during the laying period, structural bone formation stops and only medullary bone is formed; however, both structural and medullary bones continue to be resorbed [88]. This intense bone loss results in osteoporosis in caged laying hens, a common bone metabolic disease leading to bone fragility [4,89].

Medullar bone, which is characteristic only for female birds starting to lay eggs, is a secondary bone tissue developing in the marrow cavity, responsible first of all for mineralization of eggshell. Its matrix is different from that of cortical bone [90,91]. On the other hand, osteoclasts from medullary bone accelerate the supply of calcium during the egg-laying cycle and for this reason, it is during this period that the most rapid bone turnover is observed [90,92]. Analysis of other bone parameters proved intensive bone turnover and effective anti-osteoporotic action of AKG in the medullary bone of the laying hens. In trabecular bone, the value of fractal dimension was the same in both groups, irrespective of treatment, and was similar to that observed in control medullary bone, which was lower compared to the value of the fractal dimension of medullary bone in AKG supplemented hens. A smaller fractal dimension in cancellous bone and control medullary bone indicates greater bone loss, while a greater fractal dimension of medullary bone after AKG supplementation indicates a reduced bone loss. Fractal dimension is a parameter that shows structural bone changes and is used to describe irregularities in bone structure, as an indicator of osteoporosis [93].

The analysis of collagen content in the cartilage and various types of bone indicated increased bone remodeling in the AKG supplemented hens in the current study. Furthermore, COMP was found to be expressed abundantly in articular cartilage and is known to promote fibrillogenesis of type I and type II collagens. Articular COMP analysis in the AKG supplemented hens in the current study was consistent with data from collagen distribution, which showed an increased presence of immature collagen, indicating enhanced fibrillogenesis in hens at the age of 60 weeks.

## 5. Conclusions

The current study showed that dietary AKG supplementation at 1% concentration for 29 weeks during the laying cycle significantly decreased the cholesterol content of breast muscle, which is beneficial for consumers, and showed an anti-osteoporotic action, improving cancellous and medullary bone parameters, increasing bone collagen synthesis and enhancing immature collagen content, and promoting fibrillogenesis in articular cartilage.

## Figures and Tables

**Table 1:** Composition and nutrient content of experimental diet, g/kg air-dry matter.

Ingredients	Content
Ingredient (g/kg)	
Corn	422.10
Wheat	210.00
Soybean meal	236.00
Rapeseed oil	20.00
Limestone	90.00
Monocalcium phosphate	12.50
NaCl	3.00
DL-Methionine	1.40
Vitamin-mineral premix ^1^	5.00
Nutrients composition	
Metabolizable energy, MJ/kg ^2^	11.60
Crude protein	170.00
Lys	8.35
Met	4.10
Ca	37.00
Total P	6.15
Available P	3.90

^1^ The premix provided per 1 kg of diet: vitamin A, 10,000 IU; vitamin D3, 3000 IU; vitamin E, 50 IU; vitamin K3, 2 mg; vitamin B1, 1 mg; vitamin B2, 4 mg; vitamin B6, 1.5 mg; vitamin B12, 0.01 mg; Ca-pantotenate, 8 mg; niacin, 25 mg; folic acid, 0.5 mg; choline chloride, 250 mg; manganese, 100 mg; zinc, 50 mg; iron, 50 mg; copper, 8 mg; iodine, 0.8 mg; selenium, 0.2 mg, cobalt, 0.2 mg.; ^2^ Calculated according to European Table [36] as a sum of the ME content of components.

**Table 2:** The influence of alpha-ketoglutarate (AKG) supplementation on daily feed intake during the experimental period, 31–60 weeks of age, and bodyweight of laying hens at the age of 60 weeks.

Parameter	CONT	AKG	*p*-Value
Initial body weight, kg	1.80 ± 0.03	1.88 ± 0.04	*0.073*
Final body weight, kg	1.95 ± 0.03	2.05 ± 0.04	0.065
Daily feed intake, g	118.7 ± 1.2	116.6 ± 1.1	0.226
Laying rate, %	95.8 ± 0.45	96.6 ± 0.377	0.209

Data are expressed as mean ± standard error. The CONT group included laying hens not supplemented with AKG; the AKG group included laying hens supplemented with AKG at a dose of 1.0% (10 g/kg feed).

**Table 3:** The influence of AKG supplementation during the experimental period (31–60 weeks of age) on blood serum biochemistry in laying hens at the age of 60 weeks.

Parameter	CONT	AKG	*p*-Value
IGF-I, ng/mL	6.43 ± 0.33	7.20 ± 0.19	0.061
OPG, pg/mL	801 ± 35	696 ± 25	0.022
GH, ng/mL	1.99 ± 0.13	2.34 ± 0.19	0.126
Insulin, uIL/mL	8.70 ± 1.12	9.76 ± 0.67	0.426
BALP, ng/mL	1.61 ± 0.07	4.65 ± 0.31	<0.001
Ghrelin, pg/mL	136 ± 8	184 ± 4	<0.001
IL-1β, pg/mL	12.7 ± 1.4	13.9 ± 1.2	0.530
Leptin, pg/mL	41.0 ± 1.7	49.5 ± 3.4	0.041
OC, ng/mL	1.59 ± 0.09	1.25 ± 0.07	0.007
RANKL, ng/mL	0.541 ± 0.063	0.927 ± 0.104	0.004
TNF-α, ng/mL	19.9 ± 2.1	21.7 ± 1.6	0.489
VitD, pg/mL	312 ± 16	276 ± 22	0.210
Cholesterol, mmol/L	3.57 ± 0.08	3.28 ± 0.06	0.009

Data are expressed as mean ± standard error. IGF-1 – insulin-like growth factor 1; OPG—osteoprotegerin; GH—growth hormone; BALP – bone alkaline phosphatase; IL-1 β—interleukin-1β; OC—osteocalcin; RANKL—receptor activator of nuclear factor-kappa Β ligand; TNF-a—tumor necrosis factor alpha; VitD—vitamin D. The CONT group included laying hens not supplemented with AKG; the AKG group included laying hens supplemented with AKG at a dose of 1.0% (10 g/kg feed).

**Table 4:** The influence of AKG supplementation during the experimental period (31–60 weeks of age) on the breast muscle microstructure, physical properties and breast muscle cholesterol content in laying hens at the age of 60 weeks.

Parameter	CONT	AKG	*p*-Value
Absolute breast muscle weight, kg	0.190 ± 0.005	0.196 ± 0.004	0.421
Breast muscle weight of BW, %	9.76 ± 0.26	9.64 ± 0.16	0.690
pH_15min_	6.21 ± 0.04	6.20 ± 0.04	0.846
pH_24h_	5.74 ± 0.01	5.72 ± 0.01	0.153
L* (Lightness)	34.47 ± 0.27	36.49 ± 0.96	0.069
a* (Redness)	1.06 ± 0.05	0.92 ± 0.05	0.104
b* (Yellowness)	1.89 ± 0.15	2.31 ± 0.17	0.084
Chroma	2.19 ± 0.13	2.49 ± 0.18	0.194
Hue angle	30.29 ± 2.29	22.28 ± 1.09	0.012
Drip loss_24h_, %	0.224 ± 0.022	0.301 ± 0.015	0.009
Fibre diameter, μm	50.27 ± 1.39	49.59 ± 0.65	0.977
Shear force, N	23.62 ± 1.33	30.10 ± 0.91	<0.001
Cholesterol content, mg/100 g of meat	41.31 ± 3.08	29.04 ± 2.54	0.006

Data are expressed as mean ± standard error. The CONT group included laying hens not supplemented with AKG; the AKG group included laying hens supplemented with AKG at a dose of 1.0% (10 g/kg feed).

**Table 5:** The influence of AKG supplementation during the experimental period (31–60 weeks of age) on muscle fatty acid profile in laying hens at the age of 60 weeks, % of total fatty acids.

Fatty Acid	CONT	AKG	*p*-Value
C12:0	0.095 ± 0.007	0.076 ± 0.011	0.177
C14:0	0.769 ± 0.021	0.735 ± 0.028	0.345
C16:0	23.5 ± 0.2	23.9 ± 0.3	0.398
C18:0	8.79 ± 0.21	9.50 ± 0.33	0.090
C20:0	0.100 ± 0.007	0.131 ± 0.013	0.112
C22:0	0.149 ± 0.038	0.110 ± 0.012	0.342
C24:0	0.118 ± 0.008	0.143 ± 0.011	0.098
ΣSFA	33.5 ± 0.4	34.6 ± 0.5	0.118
C15:0	0.136 ± 0.006	0.129 ± 0.003	0.381
C17:0	0.306 ± 0.021	0.271 ± 0.014	0.175
Σ OCFA	0.442 ± 0.025	0.389 ± 0.020	0.114
ΣBCFA	0.098 ± 0.011	0.105 ± 0.010	0.645
C14:1c9	0.065 ± 0.006	0.066 ± 0.009	0.926
Σ C15:1	4.78 ± 0.22	5.12 ± 0.45	0.505
C16:1c7	0.821 ± 0.024	0.813 ± 0.033	0.842
C16:1c9	1.76 ± 0.10	1.72 ± 0.09	0.768
C17:1 c9	1.10 ± 0.06	1.17 ± 0.09	0.490
C18:1c9	27.8 ± 0.7	27.4 ± 1.0	0.751
C18:1c11	2.44 ± 0.06	2.57 ± 0.06	0.132
C20:1c11	0.269 ± 0.057	0.192 ± 0.010	0.194
Σ MUFA cis	39.0 ± 0.6	39.1 ± 0.8	0.960
C18:2n-6 LA	15.1 ± 0.4	14.4 ± 0.4	0.248
C18:3n-3 ALA	1.08 ± 0.06	0.94 ± 0.7	0.181
C18:3n-6 GLA	0.152 ± 0.013	0.133 ± 0.011	0.284
C20:2n-6	0.151 ± 0.006	0.150 ± 0.012	0.941
C20:2n9	0.102 ± 0.011	0.108 ± 0.011	0.717
C20:3n6	0.413 ± 0.037	0.434 ± 0.030	0.622
C20:4n-6 AA	6.59 ± 0.41	6.47 ± 0.49	0.858
C20:5n3 EPA	0.085 ± 0.003	0.078 ± 0.008	0.707
C22:4n6	0.675 ± 0.034	0.749 ± 0.061	0.307
C22:5n6	0.224 ± 0.019	0.270 ± 0.028	0.188
C24:1n9	0.118 ± 0.007	0.131 ± 0.011	0.357
C22:5n-3 DPA	0.522 ± 0.031	0.503 ± 0.037	0.699
C22:6n3 DHA	1.59 ± 0.11	1.48 ± 0.15	0.548
Σ PUFA	26.8 ± 0.6	25.8 ± 0.4	0.209
Σ n-3	3.35 ± 0.14	2.97 ± 0.15	0.287
Σ n-6	23.2 ± 0.5	22.6 ± 0.3	0.337
Σ LC-PUFA	10.2 ± 0.6	10.1 ± 0.8	0.946
Σ LC n-6	8.00 ± 0.47	8.08 ± 0.61	0.918
Σ LC n-3	2.17 ± 0.14	2.02 ± 0.18	0.533
n-6/n-3	6.99 ± 0.29	7.78 ± 0.40	0.121
PUFA/SFA	0.799 ± 0.021	0.747 ± 0.013	0.053

Data are expressed as mean ± standard error. SFA—saturated fatty acids; OCFA—odd-chain fatty acids; BCFA—branched chain fatty acids; MUFA—monounsaturated fatty acids; PUFA—polyunsaturated fatty acids; LC—long chain. The CONT group included laying hens not supplemented with AKG; the AKG group included laying hens supplemented with AKG at a dose of 1.0% (10 g/kg feed).

**Table 6:** The influence of AKG supplementation during the experimental period (31–60 weeks of age) on general, geometric and mechanical (structural and material) properties of tibia in laying hens at the age of 60 weeks.

Parameter	CONT	AKG	*p*-Value
Bone weight, g	11.87 ± 0.11	11.68 ± 0.25	0.525
Tibia bone weight of BW, %	0.610 ± 0.010	0.576 ± 0.016	0.091
Bone length, mm	121 ± 1	122 ± 1	0.547
Seedor index, mg/mm	98.1 ± 1.3	95.7 ± 1.7	0.341
MRWT	0.350 ± 0.026	0.363 ± 0.031	0.738
Cortical index, %	25.5 ± 1.4	26.0 ± 1.6	0.805
CSA, mm^2^	18.2 ± 0.8	19.0 ± 1.2	0.586
CSMI, mm^4^	78.8 ± 4.1	85.4 ± 6.6	0.707
Yield load, N	94 ± 4	98 ± 6	0.552
Elastic work, mJ	18.6 ± 1.5	19.5 ± 1.6	0.700
Fracture load, N	171 ± 6	176 ± 7	0.794
Work to fracture, mJ	121 ± 6	124 ± 7	0.696
Stiffness, N/mm	259 ± 9	270 ± 13	0.495
Young’s modulus, GPa	7.94 ± 0.38	7.54 ± 0.48	0.523
Yield strain, %	0.615 ± 0.021	0.650 ± 0.028	0.322
Yield stress, MPa	49.0 ± 3.4	48.8 ± 3.6	0.665
Fracture strain, %	1.92 ± 0.05	1.97 ± 0.07	0.610
Fracture stress, MPa	94.0 ± 6.3	87.7 ± 5.5	0.840
BMD, g/cm^2^	0.245 ± 0.021	0.227 ± 0.026	0.611

Data are expressed as mean ± standard error. MRWT—bone diaphysis mean relative wall thickness; CSA—bone diaphysis cross-sectional area; CSMI—bone diaphysis cross-sectional moment of inertia; BMD—bone mineral density. The CONT group included laying hens not supplemented with AKG; the AKG group included laying hens supplemented with AKG at a dose of 1.0% (10 g/kg feed).

## Supplementary Materials

The following are available online at https://www.mdpi.com/2076-2615/10/12/2420/s1, Table S1: Composition and nutrient content of pre-experimental, pre-laying diet, g/kg dry matter, Table S2: The calculated amino acids content in the experimental basal diet for laying hens.
