# Supplementary material for: Alpha-Ketoglutarate: An Effective Feed Supplement in Improving Bone Metabolism and Muscle Quality of Laying Hens: A Preliminary Study
Source: Animals (Basel). 2020 Dec 17;10(12):2420. doi: 10.3390/ani10122420 (PMC7767309; doi:10.3390/ani10122420)
Supplement: Supplementary file 1 [file animals-10-02420-s001.pdf]

Supplementary material

Table S1. Composition and nutrient content of pre-experimental, pre-laying diet, g/kg dry matter.

Ingredients	Content
Ingredient (g/kg)	
Corn	320
Wheat	353
Soybean meal	220
Alfalfa meal	20.0
Rapeseed oil	6.00
Limestone	53,5
Monocalcium phosphate	13.0
NaCl	3.00
DL-Methionine	1.50
Vitamin-mineral premix[1]	10.0
Nutrients composition	
Metabolizable energy, MJ/kg[2]	11.50
Crude protein	175.00
Lys	8.40
Met	4.20
Ca	22.5
Available P	4.10

[1]The premix provided per 1 kg of diet: vitamin A, 10,000 IU; vitamin D3, 3,000 IU; vitamin E, 50 IU; vitamin K3, 2 mg; vitamin B1, 1 mg; vitamin B2, 4 mg; vitamin B6, 1.5 mg; vitamin B12, 0.01 mg; Ca-pantotenate, 8 mg; niacin, 25 mg; folic acid, 0.5 mg; choline chloride, 250 mg; manganese, 100 mg; zinc, 50 mg; iron, 50 mg; copper, 8 mg; iodine, 0.8 mg; selenium, 0.2 mg, cobalt, 0.2 mg.; [2]Calculated according to European Table [33] as a sum of the ME content of components.

Table S2. The calculated amino acids content in the experimental basal diet for laying hens.

Amino Acid	Content, g/kg
Lysine	8.35
Methionine	4.10
Methionine + Cysteine	7,2
Tryptophan	2.0
Threonine	6.3
Isoleucine	4.5
Leucine	7.1
Valine	14.5
Phenylalanine + Tyrosine	8.0
